# Dreaming and insight

**DOI:** 10.3389/fpsyg.2013.00979

**Published:** 2013-12-24

**Authors:** Christopher L. Edwards, Perrine M. Ruby, Josie E. Malinowski, Paul D. Bennett, Mark T. Blagrove

**Affiliations:** ^1^Department of Psychology, Swansea UniversitySwansea, UK; ^2^Brain Dynamics and Cognition Team, Lyon Neuroscience Research Centre, INSERM U1028Lyon, France; ^3^Department of Psychology, University of BedfordshireLuton, UK

**Keywords:** Dream, sleep, REM sleep, insight, psychotherapy

## Abstract

This paper addresses claims that dreams can be a source of personal insight. Whereas there has been anecdotal backing for such claims, there is now tangential support from findings of the facilitative effect of sleep on cognitive insight, and of REM sleep in particular on emotional memory consolidation. Furthermore, the presence in dreams of metaphorical representations of waking life indicates the possibility of novel insight as an emergent feature of such metaphorical mappings. In order to assess whether personal insight can occur as a result of the consideration of dream content, 11 dream group discussion sessions were conducted which followed the Ullman Dream Appreciation technique, one session for each of 11 participants (10 females, 1 male; mean age = 19.2 years). Self-ratings of deepened self-perception and personal gains from participation in the group sessions showed that the Ullman technique is an effective procedure for establishing connections between dream content and recent waking life experiences, although wake life sources were found for only 14% of dream report text. The mean Exploration-Insight score on the Gains from Dream Interpretation questionnaire was very high and comparable to outcomes from the well-established Hill (1996) therapist-led dream interpretation method. This score was associated between-subjects with pre-group positive Attitude Toward Dreams (ATD). The need to distinguish “aha” experiences as a result of discovering a waking life source for part of a dream, from “aha” experiences of personal insight as a result of considering dream content, is discussed. Difficulties are described in designing a control condition to which the dream report condition can be compared.

## Introduction

There have been many claims of dreams enabling individuals to obtain insight about their emotional and interpersonal life (e.g.,Freud, 1953/1900; Rycroft, 1979; Blechner, 2001). However, much of the evidence here is anecdotal, or based on case-studies, and scientific investigation is difficult. There have also been claims about scientific discoveries being derived from dream experiences (Barrett, 1993), for example, that Kekulé's dream or vision of “whirling snakes” led him to insight regarding the structure of the Benzene ring, and that a dream of Mendeleev led him to his structure for the periodic table of the elements (Barrett, 2001, with critical account by Baylor, 2001). Whilst it is difficult to validate claims that some scientific insights are attributable to dream experiences, numerous cognitive experiments have been devised to assess the relationship between sleep and insight. Wagner et al. (2004) used a mathematical task, the number reduction task (NRT), which can be solved either by a relatively lengthy calculation method, or by more sudden insight into a hidden rule. In the study initial training on the NRT was followed by 8 h of either nocturnal sleep, or nocturnal wakefulness, or daytime wakefulness. At subsequent retesting, more than twice as many subjects gained insight after sleep than after wakefulness. The authors concluded that sleep, by restructuring new memory representations, facilitates extraction of explicit knowledge, and results in insightful behavior.

Later studies, investigating the cerebral correlates of cognitive insight using the NRT and fMRI, highlight an involvement of the prefrontal cortex in the insight process. Rose et al. (2010) found that insight was associated with fMRI-signal increase in the ventral striatum and the right ventrolateral prefrontal cortex. Following this, Darsaud et al. (2011) identified the neural precursors of delayed insight on this task. In their study, normal volunteers practiced the NRT during two fMRI sessions (training and retest), taking place 12 h apart across a night of sleep. After this delay, some subjects gained insight into the hidden task structure (termed solvers), whereas the others did not (termed non-solvers). It was found that, during training, solvers and non-solvers differed in their cerebral responses associated with implicit learning. Furthermore, in solvers, but not in non-solvers, response patterns were also transformed overnight, with enhanced responses in ventral medial prefrontal cortex, an area previously implicated in the consolidation of declarative memory. During retest in solvers, before they gained insight into the hidden rule, significant responses were also observed in the same medial prefrontal area.

As there is experimental support for the hypothesis that sleep facilitates cognitive insight and creative thinking (Maquet and Ruby, 2004; Stickgold and Walker, 2004; Wagner et al., 2004; Darsaud et al., 2011), it is plausible that sleep could allow personal/affective insight, and that this might be reflected in dream content, given the interaction between characters, and emotional content, in most dreams (Nielsen and Lara-Carrasco, 2007). Interestingly, the ventral medial prefrontal cortex, whose activity is modified during sleep and is enhanced before cognitive insight during wakefulness (Darsaud et al., 2011), shows increased activity in REM sleep compared to NREM sleep (for a review, see Ruby, 2011). As a consequence, one may expect that REM dreams could be more supportive of insight than NREM dreams. In support of this hypothesis, Walker et al. (2002) reported that REM awakenings provide a significant advantage in the number of anagrams solved compared with NREM awakenings, the number solved being equal to that of wake time trials. The authors concluded that the neurophysiology of REM sleep represents a brain state more amenable to flexible cognitive processing than is NREM sleep. A similar conclusion is drawn by Cai et al. (2009), who found that, in comparison to quiet rest and NREM sleep, REM sleep enhances the integration of unassociated information for creative problem solving. Related to this, Stickgold et al. (1999) found that subjects awakened from REM sleep show greater priming by weak primes (e.g., thief-wrong) than by strong primes (e.g., hot-cold). In contrast, strong priming exceeds weak priming in NREM sleep, just as in wake. The authors conclude that the shift in weak priming seen after REM sleep awakenings suggests that cognition during REM sleep is qualitatively different from that of waking and NREM sleep and may reflect a shift in associative memory systems. They hypothesize that this shift underlies the bizarre and hyper-associative character of REM sleep dreaming, and that these cognitive and dream content characteristics of REM sleep are due to the cholinergic and noradrenergic neuromodulation specific to that sleep stage.

Another neurochemical change, the increase in cortisol in the second half of sleep, is proposed by Payne (2010) as being one of the reasons for the increase in bizarreness of dreams across the night, which she says may be reflective of hyper-associativity, and of creative thinking and novel combinations of previous information, although Blagrove et al. (2012) show that this increase in cortisol occurs for NREM and REM sleep. It may also be that the relationship of sleep to insight is a component of the widely evidenced view that sleep, and in particular REM sleep, is involved in emotional memory consolidation and emotional regulation (e.g., Walker and Van der Helm, 2009; Perogamvros and Schwartz, 2012; Groch et al., 2013). This memory consolidation during sleep is held to go beyond the making permanent of individual items, to involve also a process of abstraction from experience, and the integration of new experiences into current schemata, so that the gist of experiences is distilled (Lewis and Durrant, 2011; Stickgold and Walker, 2013). Whereas Lewis and Durrant (2011) and Diekelmann and Born (2010a,b) conclude that current empirical knowledge points more to such abstraction and schemata production occurring in slow wave sleep, Stickgold and Walker (2013) state that not enough is known to exclude REM from these processes, and that evidence of the neural replay of waking life events during REM sleep (Louie and Wilson, 2001) supports the inclusion of REM in these processes.

Such neural processes of insight and memory consolidation during sleep are proposed by Wagner et al. (2004) and Wamsley and Stickgold (2011) to be related to the formal characteristics of dream experiences. As stated by Stickgold et al. (2001), “dreams would appear to be not only unpredictable and bizarre but highly emotional as well” and they propose that “these features reflect an attempt, on the part of the brain, to identify and evaluate novel cortical associations in the light of emotions mediated by limbic structures activated during REM.”

An important precursor, however, to the claim that dreams are related to these neutrally-based insight or schemata changing processes, or even that dreams themselves, when considered during wakefulness, can lead to insight, is that dream content reflects, or is relatable to previous waking life experiences. There is indeed considerable evidence for this, with dream content being related to waking life concerns or events in naturalistic studies (Saredi et al., 1997; Domhoff, 2003; Schredl, 2006; Bulkeley and Domhoff, 2010; Blagrove et al., 2011a,b; Malinowski and Horton, 2011), to concerns that the dreamer has been instructed to dream of, in comparison to control concerns (Nikles et al., 1998), and to thoughts consciously suppressed prior to sleep (Wegner et al., 2004; Bryant et al., 2011). Furthermore, individuals who have experienced a trauma can be differentiated from control individuals on the basis of dream reports (Hartmann et al., 2001a,b; Kuiken et al., 2008). Although there are caveats here, of dreams being generic (Hobson and Kahn, 2007), and of studies in which individuals with prominent waking life characteristics cannot be distinguished from controls on the basis of dream reports (paraplegia and deaf-mutes, in Voss et al., 2011; blindness, in Staunton and O'Rouke, 2012), overall the findings are of dream content being linked to waking life experience. Whether the manner of representation of waking life in dreams allows for creative insight about waking life is then the question to be addressed. The null-hypothesis is that dreams do not provide novel or insightful information to the dreamer, and nor does dream content provide useful information about the dreamer to anyone else; as stated by the psychiatrist Allan Hobson: “I never learned anything from a client's dreams that I did not already know.” (Hobson and Schredl, 2011).

Contrary to this null-hypothesis, there have been many claims of the clinical use of dreams providing personal insight. According to Pesant and Zadra (2004), in therapy “three types of gains are generally described as a result of dream interpretation: (1) client insights, (2) increased involvement of the client in the therapeutic process, and (3) a better understanding of clients' dynamics and clinical progress.” To evidence point (1), they cite researchers and clinicians' reports that the clinical use of dreams enhances clients' insight, with better self-knowledge, self-understanding, or self-awareness. In order to define and characterize insight they refer to Elliott's et al.(1994) conceptualization of it “as containing four elements: (1) metaphorical vision—to see oneself in a new light; (2) connection—to uncover some patterns in one's existence or some links between different aspects of one's experience; (3) suddenness—an affective reaction of surprise, as if things fell down into place in one's mind; and (4) newness—the sense of having discovered something that was not previously known.”

The first of these four elements is explored by Lakoff (1993), who proposes that waking life concerns are represented metaphorically in dreams (the view also of Domhoff, 2003), and that the production of dreams, and the interpretation of dreams, draw upon our waking life system of conceptual metaphors. Hartmann (1998) similarly proposes that dreams portray waking life emotional concerns as picture metaphors, so as to interconnect old and new memories. For Hartmann, this has the function of ameliorating recent distressing emotions, a function that would hold for dreams that are recalled on waking as well as for ones that are not recalled. He thus proposes that dreaming, even where dreams are not recalled, acts as psychotherapy (see also Hartmann, 1995). Such proposals of metaphor production in dreaming can be seen as a special case of the general issue of metaphors in therapy, as described by Evans (1988). For Evans (1988), metaphors can originate from the client or the therapist, and he gives examples where the interpretation or discussion of a metaphor, which can lead to progress and insight, is aided by the “emotional distance” from threatening material that the metaphor allows. Pesant and Zadra (2004) make a similar observation that clients may be less reluctant to discuss disturbing issues when these are approached through dream exploration, because of the safer distance between the client and the material evoked by a dream. Similarly, Rennie (1994) suggests that narratives allow patients in psychotherapy to talk around their problems, but that gradually sources of personal concern are brought to light, and in this way narratives help create the possibility of insight.

In a metaphor, the tenor, or topic, is expressed in terms of the vehicle, which can be more familiar or more concrete. Evans (1988) holds that using metaphor to describe one's experience catalyses change and growth, and advocates a conscious use of metaphors for this, because “the language of inner experience and self-discovery is largely metaphorical.” He states that, for tenor and vehicle, “this interaction between the two systems of associations extends the meaning of both terms, which contributes to the epistemic creativity of metaphor.” Similarly, Tourangeau and Sternberg (1982) propose that this interaction enables new things to be discovered, and not just for the two concepts being associated but for the whole of their domains as well. Furthermore, Tourangeau and Rips (1991) describe metaphor interpretation as being more reliant on the perception of emergent, rather than well-established, similarities between the two concepts being compared, such that metaphor creates an emergent feature, which is a novel insight into the two previously unrelated concepts.

For Evans (1988), becoming aware of and exploring one's metaphors, including one's novel metaphors, is part of becoming more psychologically minded, and is especially important for life metaphors, which, he claims, are often unconscious, involving tacit knowledge of one's conscious and social experience. Considering the metaphorical content of dream reports would involve processes similar to Kopp and Craw's (1998) 7-step interview protocol for exploring and transforming client-generated metaphors, which they propose aids the accessing and transforming of tacit knowledge.

These accounts of metaphor provide a theoretical justification for the proposal that examining dream content can result in personal insight. Lakoff (1993) emphasizes that each person has many conceptual metaphors that they do not have conscious access to, nor control over. He argues that cognitive and linguistic activity relies upon cognitive links which are created because of aspects of similarity between idioms or concepts. This allows the mapping of characteristics of one idiom or concept onto another, creating “conceptual metaphors.” These metaphors are claimed by Lakoff and Johnson (1980) to be very influential in human understanding of the world, and as easily identifiable in everyday language. One example they give of a common linguistic metaphor within the English language is the “LOVE IS A JOURNEY” metaphor (Lakoff and Johnson, 1980; Lakoff, 1993). This metaphor can be detected in descriptions such as “the relationship is running into trouble,” “the relationship is on the rocks,” and “we must go our separate ways,” because romantic attachment is being described in terms of physical movement. Lakoff (1993) proposes that cognition during dreaming likewise involves the mapping of abstract concepts onto physical concepts. He suggests that metaphorical understanding of a dream is possible without personal information about the dreamer, but that information about the dreamer allows metaphors specifically relevant to the dreamer's life to be interpreted, even though, in some instances, the dreamer, upon waking, does not know whether anything from waking life is being expressed metaphorically by the overt content.

However, whereas the link between dream content and metaphor has been frequently proposed (e.g., Ullman, 1969; Antrobus, 1977; Lakoff, 1993), and the ubiquity of the use of metaphors in thinking has been described by Lakoff and Johnson (1980), there is now debate about the extent of empirical evidence for an embodied basis for conceptual metaphors (Shapiro, 2011). For the purposes of the current paper, though, it is the ubiquity of metaphorical thought that is important rather than the derivation of basic concepts from direct physical experience, which Shapiro criticizes. There has also been a criticism of Lakoff by Glucksberg and McGlone (1999), who state that they “find no evidence that people automatically and invariably draw upon fixed sets of domain-to-domain mappings in order to interpret metaphors.” They thus propose a “minimalist” account of metaphor production and understanding, where the metaphor vehicle will often be a prototypical member of an attributive category; the attributive category is thus applied to the metaphor topic. They contrast this with Lakoff and Johnson's (1980) multiple mappings account, in which there are systematic mappings between topic and vehicle, derived from conceptual knowledge. Interestingly, for the current paper, Glucksberg and McGlone's (1999) simpler account of the cognition required for metaphorical thinking might be taken as more relevant to the levels of cognition that dreaming may be capable of, and hence as according with Antrobus' (1977) account of metaphor in dreams.

## Dream appreciation

To test the claim that insight can follow the consideration of dream content, the current study uses the “Dream Appreciation” technique devised by Ullman (1996). There has been work by DeCicco (2007a) on correlations between dream content measures and outcome measures that result from use of an earlier version of the Ullman technique as described in Ullman and Zimmerman (1979). DeCicco (2007a) concluded that participants found many connections between waking life and dream content, with “discovery” experiences originating from the technique. However, mean outcome scores were not reported. To our knowledge the current study is the first to report group mean outcomes of the standard (Ullman, 1996) technique, apart from a study summarized in a short report by Blagrove et al. (2010). In that study there were 13 participants (4 males, 9 females; all undergraduate students), 22 dreams were reported, and each group session lasted from 45 to 75 min. After the group a Visual Analogue Scale (VAS), anchored 0 = not at all and 100 = very much, was used for participants to rate the degree to which the session gave them any new understanding of what the dream means: here, mean rating on the VAS for this new understanding about the dream variable = 67.8 (*SD* = 18.2). Another VAS, also anchored 0 = not at all and 100 = very much, was used for participants to respond to the question “Did the session for this dream give you new understanding or insight about yourself or about any aspect of your life?” Here, mean rating on the VAS for this new understanding about yourself variable = 57.0 (*SD* = 15.5). Support was thus given for the usefulness of the Ullman technique.

## Ullman dream appreciation procedure

One of the researchers (MB) has extensive experience of conducting dream appreciation groups that follow the procedure devised by Ullman (1996), and has received training from a leading associate and colleague of Montague Ullman. The procedure involves the following stages: (1A) reading of the dream aloud by the dreamer; (1B) clarification of the dream report by the group asking questions of the dreamer; (2A) brief discussion of the dream by the group members other than the dreamer so as to imagine what feelings they would have experienced if the dream were their own, and then, (2B), eliciting these individuals' projections about the dream in terms of their own lives so as to give symbolic or metaphorical meaning to the dream images; (3A) response by the dreamer to stage (2); (3B.1) description by the dreamer of his/her waking life context for the dream, in terms of the dreamer's life experiences, with particular emphasis on recent experiences and concerns; (3B.2) reading the dream back to the dreamer, in the second person, so that any additional information about the dream or waking life can be obtained; and (3B.3) orchestration, in which all members of the group suggest connections between information that the dreamer has given about their dream and information the dreamer has given about the dreamer's life. For a full description of the process, see Ullman (1996). The technique can be compared to Hill's (1996) Exploration-Insight-Action model of dream interpretation: Ullman's stages 1A and 1B and 3B.1 are similar to the first stage of Hill's model, in which exploration of the dream occurs through the dreamer telling the dream, reporting feelings from the dream, and providing associations to the dream images, so as to find waking life sources of the dream. Stage 2 and the dreamer's response to that stage would occur when the Hill model is used in a group (Hill, 1996; Hill, Chapter 11). Ullman's stage 3B.3 has similarities with Hill's Insight stage, in which possible meanings of the dream are explored. The Ullman procedure thus mainly maps onto the first two stages of the Hill technique, and does not map onto the Hill model's third, Action stage, in which behavioral or cognitive changes as a result of the dream are considered. It should be noted that such a restriction to the first two Hill stages is also present in much clinical work with dreams. As stated by Hill and Knox (2010, p. 9): “although both cognitively and psychoanalytically oriented therapists used many activities to work with dreams, they most often focused on exploring and understanding the dreams; they rarely addressed how clients might change their dreams or make changes in waking life based on their understanding of dreams.”

The Ullman technique thus relies on the identification by participants of waking life sources of dream content, such as recent events or concerns, with the non-directive assistance of the other group members. According to Hill et al. (1998), the use of association allows clients to make “previously unconsidered connections between dream images and other thoughts and memories,” and they conclude that solely describing a dream, without producing associations, requires the client to stay within the dream, with less likelihood of identifying “engagingly novel thoughts.” In Hill's method the depth and accuracy of inferences from dream images are dependent on the work carried out exploring the possibility of associations between dream content and waking life experiences, and increases in insight are found with more time spent in this association stage (Hill et al., 1998).

However, in the eliciting of associations Grünbaum's (1984) tally argument does have to be borne in mind. This is the critique that even if a dreamer states that an item of dream content is related to some aspect of waking life, there is no evidential value to this belief of the client. Several aspects of the Ullman technique have the aim of reducing the forcefulness of suggestions from group members of relationships between dream content and recent waking life experiences that the dreamer has described, while keeping to the purpose of holding a group, which is that group members may identify valid or plausible connections, not hitherto recognized by the dreamer, between the dream report and the dreamer's recent life experiences. Obviously there may be an experimenter demand in the search by participants for memory sources of dreams, and in their consideration of how plausible possible memories are as sources. However, although this may operate for some decisions regarding sources, the empirical literature does support the claim that dreamers can, when awake, identify memory sources of dream content (Nielsen and Stenstrom, 2005; Blagrove et al., 2011a,b), and categorize waking life associations into episodic and semantic memory sources (Baylor and Cavallero, 2001; Fosse et al., 2003). Also, a more experiential counter to Grünbaum's argument can be found in Blass (2002, pp. 169–188).

Kuiken et al. (2006) have differentiated between types of dreams (e.g., transcendental, existential, mundane, nightmares) and related this factor to level and type of insight following the dream. In contrast, the current study involves the participant recalling their most recent dream, whatever its content and perceived impact or lack of impact. Furthermore, whereas Kuiken has also investigated type of dream and level of subsequent insight as a function of previous experience (e.g., loss vs. trauma; Kuiken et al., 2008), for the current study a participant's waking life background is not known to the experimenters prior to any mention of it in the group session.

The Dream Appreciation technique is used with the intention of providing a way for an individual to connect the self as experienced in the dream with the self as perceived and as experiencing in waking life (Ullman, 1996). The potential for personal change within this context is as an adaptation of conscious perspective. In contrast, Hill's (1996) Exploration-Insight-Action model uses dreams in a therapeutic context, with an Action stage, in which an individual can be encouraged or challenged to change behavioral or cognitive processes after obtaining insight from working with a dream. In the Action stage clients determine what they would like to do differently, “in the dream, in life, or in continued work on issues raised by the dream” (Hill et al., 1998). Change can therefore be actively pursued by a therapist using Hill's (1996) method. In contrast we chose the Ullman Dream Appreciation method as it is appropriate for lay people to use in a non-therapeutic setting, using the manual (Ullman, 1996) as a guide, and because it has been used worldwide in various lay settings. Ullman (1996) argues that there are also structural differences between formal therapy and his Dream Appreciation technique. He asserts that there is a hierarchical structure in therapy, in which the therapist acts more as an authority figure compared to the leader of a Dream Appreciation group. The emphasis in the Dream Appreciation format is on the dreamer having overall control during the process, the leader of the group is seen more as a safety supervisor and a guide through the stages rather than a controlling influence. The individual who offers the dream regulates the information that is shared with the group and holds the power to decide whether anything discussed during the dream discussion session is relevant or useful.

## Hypotheses

In the current study, in order to assess outcomes from the Dream Appreciation technique, participants rate their Exploration-Insight gains, Experiential gains, and Action gains using the Gains from Dream Interpretation questionnaire (Heaton et al., 1998), and complete a Deepened Self-Perception questionnaire (Kuiken, 1995). We hypothesize the following:

1. Participants would rate Exploration-Insight gains and Experiential gains of the group procedure highly, and, specifically, as highly as those found by participants who follow, with a psychotherapist, the more widely researched Hill (1996) method.
2. As the Hill method has an aim of therapeutic change, whereas this is not a specific aim of the Ullman method, the participants would rate Action gains of the Ullman procedure as lower than the Exploration-Insight or Experiential gains, and as lower than occurs for the Hill method.
3. As Hill et al. (2001) found that the level of individuals' Own positive Attitude Toward Dreams (ATD) was significantly related to the Exploration-Insight gains subscale score of the Gains from Dream Interpretation (GDI) questionnaire, there would be a significant positive correlation between the Own Attitude Toward Dreams subscale of Cernovsky's (1984) ATD questionnaire, completed prior to the group, and the Exploration-Insight gains subscale of the GDI questionnaire, completed after the group.

We also conduct an exploratory analysis of the amount of text of the dream reports for which memory sources can be identified by the dreamer, with the aim of comparing our results with those of a previous study (Saredi et al., 1997) where a far less lengthy consideration of each dream occurred.

## Comparison conditions

The current dream appreciation study does not include a control condition, and so it is not possible to determine the role of dream content in the group process, or to disentangle whether any insights or gains during the group are dependent on the dream content, or solely on the group process. The current study aims to pilot how the effects of considering dream content in an Ullman Dream Appreciation group can be measured, with future studies then intended to include a control comparison. The issue of a control can be illustrated by reference to Timpanaro (1974/1985, pp. 29–45), who analyses an account by Freud of how, by free-association, a slip of the tongue of a missing word (*Aliquis*) in a Latin quotation can be linked to a current concern of the person who was conversing with him and who made the slip. Crucially Timpanaro shows how other words from the quotation, for which there were not slips of the tongue, can also be linked by free-association to the same concern, thus showing for us the importance of control conditions to test that an item of dream content can be linked to putative insight. Control conditions were used in the study of dream interpretation by Hill et al. (1993): participants who were involved in interpreting their own dreams rated sessions as deeper and as producing more insight than did participants who were involved in interpreting another person's dreams or interpreting their own recent troubling events. Hill et al. (1993) thus concluded that the efficacy of dream interpretation does not seem to be solely due to projection or to the process of interpreting any text, whether of a dream or a text that is not of a dream. However, in Diemer et al. (1996), therapist and client assessments of client insight did not differ between a session of dream interpretation and a session of interpretation of a recent troubling interpersonal event. Each session lasted 50 min and the dream or event was the focus of the session for at least 40 min. The only control condition in a study using the Gains from Dream Interpretation questionnaire, which is used in the current study, is the focussing on recent loss condition in Hill et al. (2000), where Exploration-Insight for that event condition was significantly lower than for the dream condition.

For the loss condition in Hill et al. (2000), “therapists began by asking clients to explore an aspect of their loss by describing it in detail, associating to thoughts about loss, and exploring their feelings related to the loss. During the insight stage, therapists helped clients construct new understandings of their thoughts, feelings and behaviors in relation to their loss.” However, a problem with such control conditions is that they cannot be used in a double-blind manner. This is a concern because Morewedge and Norton (2009) have shown that individuals from both Eastern and Western cultures believe that dreams contain hidden truths and consider dreams to provide more meaningful information about the world than do similar waking thoughts; this indicates that a control condition, unless the justification or theory behind it is very plausible to participants, would be at a disadvantage in comparison to an own-dream condition. Hill et al. (1998) similarly caution that experimenter demand can occur because of the common view that dream interpretation involves making associations, a view that might not be commonly held for comparison waking life event narratives.

Although a non-dream control condition is not included in the current study, we do compare participants' mean subscale scores from the GDI questionnaire with subscale scores from participants in various studies on dream interpretation by Hill, and we compare the current study subscale scores with those from the control group in Hill et al. (2000) where participants focused on a loss they had suffered rather than on a dream. In that study a parallel form of the GDI, called the Gains from Therapy, was developed for the loss condition, with the word *dream* replaced by the word *problem*.

## Method

The study was approved by the Research Ethics Committee of the Swansea University Department of Psychology.

## Participants

Eleven participants took part in the study [10 females, one male; mean age = 19.2 years (*SD* = 0.75)]. They were all undergraduate students studying psychology and they received experiment participation credits for their voluntary participation. Individuals responded to a recruitment advertisement and were invited to a briefing session about the study, which was titled “Can ‘insight’ be obtained from dreams using the Ullman ‘Dream Appreciation’ group procedure?” At this session they completed a brief screening questionnaire, so as to assess whether they met inclusion criteria of not suffering from or having had a history of any psychological problems, and not having any personal issues that might make it difficult for them to participate in a situation involving a degree of self-disclosure.

## Materials

- The Attitude Toward Dreams questionnaire (ATD; Cernovsky, 1984). This has three subscales, (1) own attitude toward dreams, (2) perceptions of attitudes of significant others toward dreams, and (3) perceptions of attitudes of other people in general toward dreams or toward individuals who publicly discuss dreams. There are 16 items, scored as agree or disagree, the three subscales have 7, 5, and 4 items, respectively, and have scores ranging 0–7, 0–5, and 0–4, respectively.
- The Gains from Dream Interpretation questionnaire (GDI; Heaton et al., 1998). This has 13 items and uses a 9 point scale for each item (1–9, with descriptors “strongly disagree” to “strongly agree”). This questionnaire comprises 3 subscales: Exploration-Insight gains, Experiential gains, and Action gains. The Exploration-Insight gains subscale is of particular importance to the current study, and is composed of the following items:
  1. I was able to explore my dream thoroughly during the session.
  6. I learned more from the session about how past events influence my present behavior.
  7. I learned more about issues in my waking life from working with the dream.
  8. I felt like I was very involved in working with the dream during the session.
  12. I learned things that I would not have thought of on my own.
  13. I was able to make some connections between images in my dream and issues in my waking life that I had not previously considered.
  (Due to a typographical error, item 2 of the GDI questionnaire, “I learned more about what this dream meant for me personally during the session,” and which would form part of this subscale, is omitted). The Experiential gains subscale has 2 items: “During the session I was able to re-experience the feelings I had in the dream” and “I felt like I was actually reliving the dream during the session.” The Action gains subscale has 5 items such as: “Because of the session, I have more of a sense that I can change my dreams when they are frightening or bad” and “I will use things that I learned in this dream interpretation in my life.”
- Section A of Kuiken's 1995 Deepened Self-Perception (DSP) questionnaire. These items are:
  1. I feel sensitive to aspects of my life I typically ignore.
  2. My dream influenced my mood even after awakening.
  3. My dream reminded me of events that occurred in my past.
  4. My dream made me feel like changing the way I live.
  A 10 point scale was used, from 0 to 9, with descriptors “strongly disagree” to “strongly agree.”

## Procedure

At the briefing session participants were informed about what the study involves and gave written informed consent to take part. Individuals taking part in the study were encouraged to write their dreams down each morning and to decide for themselves, when a dream occurred, whether they wished to share and discuss it in the group. When participants had a dream that they wished to share, they contacted the researchers to arrange a convenient time over the following few days for convening the dream group. This follows the recommendation of Ullman that dreams that are the subject of his technique should have occurred recently, unless the group has already met many times, in which case the examination of older dreams is allowed. The dream group consisted of two researchers (CE and MB) and the participant sharing the dream; each participant came to a group only once. There were thus 11 separate group sessions, each with one participant and the two researchers. This group membership was used so as to keep the group as consistent as possible, rather than having more individuals involved. It was also necessitated by the need to have the group meet as quickly as possible after the participant had a dream, which can be difficult to timetable with several people.

For the group session, each participant had written their dream report down upon waking and brought the report with them. At the start of the session the participant completed the Cernovsky (1984) ATD questionnaire, which assesses one's personal attitude to dreams and one's perceptions of the views of others about dreams. After this the participant read out loud their dream and the Ullman Dream Appreciation procedure, as described above, was followed closely and in full for all 11 participants. Note that the design of the study did not allow the possibility of the dreamer briefly reporting any further considerations or realizations about the dream at the next meeting of the group, as only one meeting was scheduled for each participant.

After the Dream Appreciation Group the participants completed the 13 item GDI questionnaire (Heaton et al., 1998) and section A of Kuiken's 1995 DSP Questionnaire.

## Transcribing and scoring dream reports

The Dream Appreciation sessions were recorded with a digital voice recorder and then transcribed. The description of the dream as stated at the start of the session (stage 1A of the Ullman procedure) is termed here the initial dream. The initial dream description in the transcription was amended according to Antrobus' (1983) definition of words to be retained in dream reports, which are “all words in sentences or phrases in which the subject was describing something that had occurred just before waking. It excluded “ahs,” “uhms,” repeated and corrected words, and all commentary on the experience, the report, or the current status of the subject.” Next, the transcription of the dream appreciation session was used to add additional text to the initial dream, where details of the dream had been remembered or clarified during the session. This process of utilizing all information from the transcript of the session about what to include in the dream text was conducted by the first author (CE) and produces what we term the “canonical dream.” The initial version of the dream and the canonical version of the dream were then compared in terms of word lengths so as to calculate a percentage change in word length due to the session.

The canonical dream was then used so that the first (CE) and second (JM) scorers could undertake the matching of dream content to the dreamer's waking life events and experiences as recorded in the transcription of the session. The two scorers worked independently in order to identify words in the dream report that the dreamer asserted or agreed in the transcript are linked with a source from their waking life or conscious experience. Each scorer highlighted words in the canonical version of each dream that the dreamer linked with a source from their waking life. The highlighted versions of the canonical dream from the two scorers were then compared with each other so as to calculate inter-rater reliability. This was computed as the sum of the number of words that both scorers agree do derive from specific waking life experiences of the dreamer present in the transcript and which the dreamer asserts is a source of the dream content, or words that both scorers agree do not derive from any waking life experience described by the dreamer, divided by the total number of words in the canonical dream, and expressed as a percentage.

## Results

The dream group sessions lasted for as long as was needed to complete all the stages of the Ullman technique; the mean length of the sessions was 67.18 min (*SD* = 14.40). The 11 dreams initially presented by the participants in stage 1A had a mean length in words of 543.27 (min = 216, max = 1654 words, *SD* = 431.02). The canonical dreams had a mean length in words of 778.27 (min = 333, max = 1779, *SD* = 415.25). The mean percentage increase in length of dream report as a result of the group process = 43.26%.

Table 1 shows group mean values for responses to the pre-group ATD questionnaire, and the post-group GDI and DSP questionnaires. The results from the subscale analysis of the ATD questionnaire indicate that most participants in the study were positive in their own attitude to dreams, and considered that significant others and society in general are positive toward dreams.

**Table 1:** **Group (*n* = 11) mean scores for Attitude Toward Dreams (ATD) questionnaire subscales, the Gains from Dream Interpretation (GDI) questionnaire subscales, three items on the GDI questionnaire that relate to learning about one's waking life, and the four Deepened Self-Perception items**.

**Measure**	**Scale range**	**Mean**	***SD***
Own ATD	0–7	5.00	1.67
Significant others ATD	0–5	2.55	1.57
Society ATD	0–4	3.73	0.47
Exploration-Insight gains^a^	1–9	8.17^**^	0.54
Experiential gains	1–9	7.28^*^	1.94
Action gains	1–9	5.78	1.04
Exploration-Insight gains item 6^b^	1–9	7.64	1.12
Exploration-Insight gains item 7^c^	1–9	7.36	1.12
Exploration-Insight gains item 13^d^	1–9	8.36	0.92
I feel sensitive to aspects of my life I typically ignore	0–9	6.36	1.86
My dream influenced my mood even after awakening	0–9	5.64	2.98
My dream reminded me of events that occurred in my past	0–9	6.91	2.77
My dream made me feel like changing the way I live	0–9	2.73	3.04

Comparison of Exploration-Insight gains and Experiential gains with Action gains:

** *p < 0.001*,

* p < 0.05.

a Due to a typographical error, item 2 of the Gains from Dream Interpretation questionnaire, “I learned more about what this dream meant for me personally during the session,” is omitted.

b Item 6 “I learned more from the session about how past events influence my present behavior.”

c Item 7 “I learned more about issues in my waking life from working with the dream.”

d Item 13 “I was able to make some connections between images in my dream and issues in my waking life that I had not previously considered.”

Internal consistency for the GDI Exploration-Insight gains subscale was acceptable (Cronbach's alpha = 0.61). The mean rating for Exploration-Insight gains showed that participants had very high agreement that such gains had occurred. The three items on the GDI questionnaire that are part of the Exploration-Insight subscale and that refer specifically to learning about one's waking life are also reported in Table 1, and show high agreement that such personal insight occurred (items 6, 7, and 13). The mean of Experiential Gains was also high (and also with high subscale internal consistency, Cronbach's alpha = 0.84). The mean rating for Action Gains was lower, and with a low internal consistency for the subscale (Cronbach's alpha = 0.53). Matched-sample *t*-tests showed that as hypothesized the Exploration-Insight mean and Experiential mean were significantly higher than the Action mean [*t*_(10)_ = 9.79, *p* < 0.001 and *t*_(10)_ = 2.37, *p* < 0.05, respectively].

There were moderate ratings for two items on the DSP questionnaire: “I feel sensitive to aspects of my life I typically ignore,” and “My dream reminded me of events that occurred in my past.” A lower rating was given to “My dream influenced my mood even after awakening,” and a far lower rating to “My dream made me feel like changing the way I live.”

As hypothesized, the relationship between Own Attitude Toward Dreams, assessed pre-group (where higher scores indicate positive attitude), was significantly correlated with Exploration-Insight gains as assessed by the GDI questionnaire (Spearman's rho = 0.68, *p* = 0.02). No other relationships between ATD and GDI subscale scores were hypothesized, however, correlations were conducted for exploratory purposes. All other correlations between the ATD and GDI subscales were found to be non-significant; rhos ranged from −0.30 (*p* = 0.371) to 0.29 (*p* = 0.385). Although no relationships were hypothesized between the ATD subscales and the DSP items, correlations were also conducted for exploratory purposes. All of these correlations were found to be non-significant; rhos ranged from −0.57 (*p* = 0.067) to 0.46 (*p* = 0.160). All correlations between the four DSP items and the three GDI subscales were non-significant with the exception of DSP item 4 (My dream made me feel like changing the way I live) and Action gains, where rho = 0.71, *p* = 0.014. For the other correlations, rhos ranged from −0.08 (*p* = 0.826) to 0.48 (*p* = 0.137).

Words in each dream report for which dreamers identified or acknowledged a waking life memory source during the session were identified from the transcripts by two scorers working independently. The inter-rater reliability, defined as percentage of words in each canonical dream for which the scorers agreed there either was, or was not, a waking life source stated in the transcript, was 83.18% (*SD* = 4.68). The mean percentage of the text of the canonical dream reports for which a waking life source could be identified from the transcript was 18.3% (*SD* = 6.2) for scorer 1 (CE), and 10.6% (*SD* = 4.8) for scorer 2 (JM). The mean percentage for the 2 scorers combined was 14.4% (*SD* = 4.1).

The length of the initial and canonical dream reports in words, and the percentage of dream report words for which a waking life source was identified, were not significantly correlated with any ATD or GDI subscale, nor any DSP item, when a correction for multiple correlations was applied (*p* < 0.005 criterion used). For the correlations of the three dream report words measures with ATD subscales, rhos ranged from −0.36 (*p* = 0.280) to 0.65 (*p* = 0.032); for the correlations with GDI subscales, rhos ranged from −0.24 (*p* = 0.478) to 0.74 (*p* = 0.009); for the correlations with DSP items, rhos ranged from −0.32 (*p* = 0.341) to 0.37 (*p* = 0.263). The only correlation out of the 30 tested that approached the significance criterion was between the mean proportion of dream report words for which a source was identified and the GDI Experiential gains subscale (rho = 0.74, *p* = 0.009). The correlations of GDI Exploration-Insight and Action gains with percentage of dream report words for which a waking life source was identified were negligible (rhos = 0.06 and −0.03, respectively, *ps* = 0.861 and.926), and remained non-significant when percentage of words data from the two scorers were analyzed separately. The correlations of DSP items with percentage of dream report words for which a waking life source was identified were also non-significant [rhos ranged from −0.06 (*p* = 0.862) to 0.36 (*p* = 0.277)] and remained non-significant when percentage of words data from the two scorers were analyzed separately.

There were some examples in the transcripts of what appeared to be metaphorical references to waking life concerns. For example, one participant described how one of her greatest fears is not being able to have a baby. The participant shared a dream in which she frequently protects her own mother, for example she “had to like force her to run away” and provides her mother with advice to escape danger, in one such instance she says “no, we've gotta go” when the mother would have stayed in a precarious situation. The concept of parenthood maps onto acts of protection in the dream, which is connected to her waking life desire to preserve her own ability to become a mother in future, and therefore “PARENTHOOD IS PROTECTION” can be suggested as a conceptual metaphor in this dream, according to the Lakoff (1993) model.

Table 2 shows means for the GDI subscales from the current study and from all papers, to our knowledge, by Hill that report group subscale means on the GDI. The mean rating in the current study for Exploration-Insight gains of 8.17 is comparable to many of the mean scores on this subscale reported by Hill and is significantly higher than the only non-dream control condition in Hill that uses the GDI (Hill et al., 2000). Table 2 also shows, for each GDI subscale, a meta-analysis mean of all dream-interpretation conditions by Hill that assess that subscale; these means are weighted by the number of participants in each study or condition. The mean Exploration-Insight gain for the current study is not significantly different from the weighted overall mean on this subscale for the dream conditions in the Hill studies.

**Table 2:** **Gains from Dream Interpretation subscale means (and SDs) from papers by Hill, with comparison of current study with Hill studies' subscale means, and comparison of current study means with weighted subscale means of all Hill dream conditions**.

**Authors**	**Date**	**Condition^a^and sample size**	**Exploration-insight gain**	**Experiential gain**	**Action gain**
Current study		Ullman dream group. (*n* = 11)	8.17 (0.54)	7.28 (1.94)	5.78 (1.04)
Heaton et al.	1998	Therapist-facilitated, following Hill (1996). (*n* = 25)	8.03 (0.73)	7.06 (1.50)	6.98 (1.07)^**^
		Self-guided, following Hill (1996) as adapted by Hill et al. (1996). (*n* = 25)	6.61 (1.35)^**^	6.76 (1.74)	5.99 (1.11)
Hill et al.	1998	Participants elaborate on image details in dream, following only the Exploration Stage of the Hill (1996) model. (*n* = 17)	4.61 (1.71)^**^	Not reported	Not reported
		Participants provide associations to images, following only the Exploration Stage of the Hill (1996) model. (*n* = 17)	6.48 (1.35)^**^	Not reported	Not reported
		Participants elaborate on image details and provide associations. Following only the Exploration Stage of the Hill (1996) model. (*n* = 17)	5.84 (1.76)^**^	Not reported	Not reported
Zack and Hill	1998	Used Hill (1996) model. (*n* = 38)	7.67 (1.10)	7.09 (1.42)	6.34 (1.74)
Rochlen et al.	1999	Pre-study discussion of benefits of dream contemplation. Participants encouraged to use strategies to boost dream recall prior to study following Hill (1996). (*n* = 14)	7.48 (1.48)	7.00 (1.70)	6.03 (1.41)
		Involved an introduction to Hill model and participation in two sessions of the group procedure prior to study following Hill (1996). (*n* = 14)	8.04 (0.96)	6.79 (1.97)	7.01 (1.34)^*^
		Pre-study discussion of theoretical perspectives regarding counseling prior to study following Hill (1996). (*n* = 14)	7.92 (0.82)	7.32 (1.48)	6.79 (1.22)^*^
Hill et al.	2000	Dream condition, following Hill (1996). (*n* = 7)	8.08 (0.52)	7.43 (0.97)	7.54 (0.49)^**^
		Control condition: Recent loss focussed on, not a dream (*n* = 7)^b^	6.63 (0.76)^**^	6.59 (0.99)	6.12 (0.82)
Wonnell and Hill	2000	Hill (1996) followed, action stage omitted (*n* = 23)	7.88 (1.06)	Not reported	6.10 (1.24)
		Hill (1996) followed, including Action stage (*n* = 20)	7.99 (1.00)	Not reported	6.98 (1.45)^*^
Hill et al.	2001	Followed Hill (1996). (*n* = 105)	7.77 (1.20)	Not reported	Not reported
Hill et al.	2003	Participants type dream and computer-generated voice asks questions, following Hill (1996). (*n* = 39)	6.72 (1.27)^**^	Not reported	6.12 (1.73)
		Therapist empathy: Followed Hill (1996) but therapists did not offer interpretations or provide action suggestions. (*n* = 26)	7.75 (0.85)	Not reported	6.60 (1.16)
		Therapist empathy + input: Followed Hill (1996), at least one therapist interpretation and one action idea offered to participants. (*n* = 29)	7.93 (0.93)	Not reported	6.81 (1.05)^**^
All Hill studies		Weighted mean (SD) of all Hill dream conditions^c^	7.40 (1.15)	7.03 (1.56)	6.51 (1.34)

Significant difference between current study mean and Hill condition mean:

* *p < 0.05*,

** p < 0.01.

a Conditions reported used Hill (1996) one-to-one therapeutic procedure unless otherwise stated.

b A parallel form of the GDI, called the Gains From Therapy, was used for the loss condition, with the word dream replaced by the word problem.

c Weighted mean uses all Hill conditions in this Table except for the Hill et al. (2000) control condition. Means are calculated with weighting for number of participants in each condition. There were no significant differences between current study subscale means and weighted subscale means of all Hill dream conditions.

The mean Experiential gains for the current study is comparable to the means for this subscale in the papers by Hill. In contrast, our Action gains mean was significantly below many of the means for this subscale reported by Hill. The current study subscale means for Experiential and Action gains do not differ significantly from the Hill studies' meta-analysis means for these two subscales.

## Discussion

Participants gave a very high mean rating for their Exploration-Insight gains from participating in the dream appreciation group. The mean rating in the current study for Exploration-Insight gains is comparable to results from the studies by Hill, but is not significantly higher than the weighted mean on this subscale of all dream conditions in the Hill studies. Importantly, the current study's Exploration-Insight subscale mean significantly exceeds the control (focusing on loss rather than a dream) condition mean in Hill et al. (2000), as did also the dream condition in that study. The mean Experiential gains in the current study is comparable to findings from studies of the Hill model. In contrast, our Action gains mean is in some cases significantly less than that found by Hill. For all three subscales the means from the current study are not significantly different from the weighted subscale means of the dream conditions in the Hill studies.

The low Action gain accords with the low rating given to the item “My dream made me feel like changing the way I live” on the DSP questionnaire. On that questionnaire participants gave moderate agreement ratings to two items: “I feel sensitive to aspects of my life I typically ignore,” and “My dream reminded me of events that occurred in my past.” There will be conflicting views of whether the data from these two items give evidence for the occurrence of insight. This is because the former item may reflect redirected attention, rather than unprecedented insight, and the latter item does not preclude the possibility that those events are routinely remembered. Whether such state cognitive changes are components of insight is a definitional as well as empirical matter about which views will differ.

The results from the GDI accord with the conclusions of Pesant and Zadra (2004) that exploring dream content and linking that content to the dreamer's waking life can help in the achievement of insights and self-understanding. However, this was achieved even though, according to the independent judges, participants identified waking life sources for a mean of only 14.4% of dream report text. This figure is comparable to that of Saredi et al. (1997), where 17.3% of dream text was found to refer to waking life concerns. Importantly, there was no significant or appreciable correlation between Exploration-Insight gains or Deepened Self-Perception scores and percentage of dream text for which a waking life source could be determined. This illustrates that a distinction needs to be made between insight about the sources of an item of dream content (“aha, this is where that part of the dream came from”) and insight about one's waking life as a result of considering the dream (“aha, this tells me about myself”). This distinction is also made by DeCicco (2007b), with her separate questions “Does the dream have meaning for you?” and “Do you have any clear insight about your life from this dream?” We thank an anonymous referee for the suggestion that furthermore it may be that these two types of insight can be compared to the distinction between associative and metaphorical thinking, and we note that it might even be that these two types of insight following examination of dream content could have different time-courses. For example, Pesant and Zadra (2004) suggest that reflecting on dreams while awake can increase the awareness of one's feelings, but not immediately after awakening from the dream. However, although these two types of insight can and should be distinguished, there may be instances where they occur together. This is because the relationship between the metaphor in a dream (the vehicle) and its waking life source (the tenor or topic) may extend the meaning of both terms (Black, 1954-1955; Tourangeau and Sternberg, 1982; Tourangeau and Rips, 1991). It is thus arguable that any new metaphor will provide some restructuring of waking life knowledge, even if the waking life issues are known to the dreamer and are already well-considered and explored.

Metaphorical references to waking life events and concerns occurred during some of the dream appreciation sessions. We suggest that the Ullman procedure results in the identification of metaphors primarily during the orchestration stage (stage 3B.3), but that even the earlier more associative stages may aid in this process. Firstly, in stages 1A and 1B, the focus is on collecting the dream report, and recalling as much of it as possible, without the distraction of attempting to interpret or identify sources of it. In stage 2B, parts of the dream content are matched by the dream group members to their own schemata, using explicit projection. This helps to identify themes in the dream, which may match themes in the lives of the other group members. This projective process does not use only simple associations to dream images, but also the exploration of “metaphorical meaning that links the image to a relevant bit of real-life experience” (Ullman, 1996, p. 35). In stage 3 the dreamer associates to and considers events and feelings from the previous day and the days before that. However, as stated by Hill (1996, pp. 49–54) for her technique, but of relevance here, when the dreamer (and, for us, group) identify a waking life schemata that is represented in the dream, the situation may then involve metaphor rather than simple association. This is so even if it was a single associated source that was involved in exciting the schemata and leading to the formation of the dream. The concept of association alone is thus not sufficient to describe the exciting of schemata that is hypothesized to be involved in dream production, nor sufficient to describe the discovery of the excited schemata during association to the dream. Hill's conceptualization of dream formation and interpretation as involving the integration of new experiences with current schemata, and Ullman's similar view of dreams making connections between past and present “tensions,” can furthermore be linked to the sleep-dependent processes of abstraction from experience, and integration of new experiences into current schemata, as described by Lewis and Durrant (2011) and Stickgold and Walker (2013). For both Ullman and Hill dream imagery can thus be metaphorical rather than solely associative, because recent emotional waking life events excite schemata (Hill, 1996) or unresolved tensions from the past (Ullman, 1996, p. 4), which are themselves structured consequences of past experience.

Although only 14.4% of dream report text was identified as being derived from specific waking life experiences, it may be that such a small or minimal amount of text is sufficient to represent a metaphor or important connection to waking life. We thank an anonymous reviewer for noting that this finding is similar to Hartmann's work on the Central Image, or Contextualizing Image in dreams, in which it is the quality or aptness of the text that refers to waking life that is important, rather than necessarily the quantity of text (Hartmann et al., 2001a,b; Hartmann and Kunzendorf, 2005-2006).

Regarding the generalizability of our group findings to individuals examining their own dreams, either on their own, or very briefly with others, a caveat is needed that the dream appreciation sessions required approximately one hour of detailed consideration of each dream. This may explain the difference between our results and those of Schredl and Erlacher (2007). In their study 444 students and 636 online respondents answered a questionnaire about dream recall frequency and creative dreams. The former sample was of mainly psychology students recruited to a study entitled “sleep, dreams, and personality.” The latter sample was of individuals recruited to an on-line study on lucid dreaming. Despite what may have been a recruitment that favored individuals interested in dreaming, respondents reported that just 7.8% of their recalled dreams were creative. The themes of the 272 creative dreams that were reported were classed into 4 categories: artistic creativity, (non-interpersonal) problem-solving, impulses and emotional insights. Forty of the 272 creative dreams were classed as giving emotional insight. This indicates that approximately 1.2% of dreams, which are presumably not the subject of a formal group examination, produce emotional insight, for individuals who may have a positive attitude to dreaming. Contrasting this figure with the results of the present study, it may thus be that it is only with some time spent on examination of dreams [for example, approximately an hour in the current study, or 40–50 min in Diemer et al. (1996), or 13 min in Hill et al. (2013)], and possibly with the help of others that a higher level of emotional insight can be obtained from examining dreams. However, in favor of generalizability of the current results, all participants in the current study took part in order to receive course credits and thus were not required to have an especial interest in dreams, or a particular belief about dreams. The preponderance of females in our sample, though, does mean that there may have been a more positive attitude toward dreams (Schredl, 2010), and greater interest in dream interpretation (Schredl and Piel, 2008), than for a sample with more males, and this may have influenced the GDI and DSP results.

A criticism of the current dream appreciation study is that a comparison non-dream control condition was not part of the study, and control conditions should indeed be used in future studies. In addition to having conditions based on the appreciation or interpretation of texts other than dream reports, such as reports of waking life episodes, or the dream of another person, conditions based on components of the Ullman technique could also be used, to which the full Ullman procedure would be compared. Such a method of comparing to isolated components of the technique has been used by Hill in controlled studies of her model. A further criticism is that it may be that alternative methods or schools of interpreting dreams (many of which are detailed in Cushway and Sewell, 2013) might result in different gains scores from those here, whether higher or lower. And it may even be that some individuals are better attuned to identifying or utilizing free-associations. Evidence for this ability is shown by Cohen et al. (2011), who found that psychoanalysts were better able to recognize healthy adults who had experienced sibling cancer during childhood, from 5-min of spontaneous free-associating speech, than were medical students, oncologists, cognitive behavioral therapists and individuals with a history of a sibling with a cancer.

We wish to consider finally two explanations for what appears to be dream content being a source of insight. As reviewed above, there may be functional characteristics of sleep, and especially REM sleep that underlie emotional memory processing and which result in novel connections being made between memories, with this memory enhancement, and creative abstraction from experience, then being reflected in dream content. An alternative view that beneficial consequences of examining dream content are unrelated to any functional role of sleep, is proposed by Blagrove (2011). In this epiphenomenal view of dreaming, beneficial consequences of examining dream content are not related to any functional characteristic of sleep, and may instead be related to a decreased self-censorship in cognition during sleep, a view proposed by Freud (1953/1900). Although both these explanations allow for recalled dreams to be a source of insight, they differ in their ascription of whether there is any cognitive consequence to unremembered dreams. In addition, as stated in the introduction, and detailed in Blagrove (2011), the null hypothesis that dreaming is not a source of personal insight, should still be borne in mind. There are similarly different explanations for the finding that, as hypothesized, the Exploration-Insight gains in the current study correlated significantly with pre-group assessment of Own positive Attitude Toward Dreams. One explanation for the correlation is that individuals who are more positive in general toward dreams are better able to identify associations and metaphorical references to waking life in their dreams; they take the dream seriously as a possible source of new information, and thus engage in the discovery and exploration of the dream. A second explanation, however, is that individuals who are highly positive on ATD are flexible and attuned to many sources of information, including suggestions made by group members, and are willing and able to engage in relaxed, gently inquisitive discussions about their emotional and personal life. They are able to identify useful associations to various materials, whether dreams, Tarot cards or personal events, or to confabulate those associations, and acknowledge their own gains from such a group activity.

Obviously the stage of sleep that the 11 dreams reported here came from is unknown, although it would be expected that most would be from REM sleep. Whereas theoretical reasons for suggesting that REM dreams may afford more insight than NREM dreams were reviewed in the introduction, a more cognitive rather than physiological view of dreaming (such as by Foulkes, 1985 or Domhoff, 2003) would not give such weight to this distinction between stages of sleep. Furthermore, other authors argue in favor of a greater effect of SWS sleep than REM sleep on the process of cognitive insight and memory-consolidation (e.g., Yordanova et al., 2008, 2010, 2012; Born and Wilhelm, 2012). Future studies will need to address whether REM sleep, N3 (SWS), or N2 dreams result in the same or different levels of insight when dream reports from those sleep stages are considered in a dream group procedure. The consideration of REM sleep dreams was found by Cartwright et al. (1980) to reduce drop-out rates for psychotherapy in comparison to a group not subjected to awakenings and collection of dream reports in the sleep lab. However, there was no significant difference in drop-out rates between this REM dream group and a group awakened the same number of times from NREM sleep. Instead, recall and discussion of dreams, whether elicited from REM or NREM sleep, was associated with staying in psychotherapy treatment, in comparison to the group that did not sleep in the lab, and was also associated with a greater expression of affect during the psychotherapy. It may thus be that levels of insight during a dream appreciation session will not differ between REM and NREM dreams, especially if dream report length is controlled. Yet, as Cartwright et al. (1980) did not measure insight as a result of considering dreams, it remains possible that REM and NREM dreams differ in their ability to be a source of insight, even if they do no differ on the outcome measures used by Cartwright et al. (1980).

To summarize, participants reported a high level of Exploration–Insight gains following the dream appreciation group procedure. However, due to the lack of a control condition run as part of a single experiment with the dream appreciation condition, the data reported here are insufficient for determining whether dream content itself is a source of insight. There are theoretical reasons for thinking that the dream group process is more than a supportive social discussion, or placebo, due to its reliance on a dream which has been formed under brain conditions that encourage, according to some theories, an integration of new with old memories and abstraction of waking life experiences into schemata. We hope that future studies may now follow the method here, and also include the assessment of gains from non-dream report control conditions.

### Conflict of interest statement

The authors declare that the research was conducted in the absence of any commercial or financial relationships that could be construed as a potential conflict of interest.
